# Available and novel plant-based carbon dots derived from Vaccaria Semen carbonisata alleviates liver fibrosis

**DOI:** 10.3389/fmolb.2023.1282929

**Published:** 2023-12-04

**Authors:** Yafang Zhao, Ertong Dai, Liyang Dong, Jinye Yuan, Yusheng Zhao, Tong Wu, Ruolan Kong, Menghan Li, Shuxian Wang, Long Zhou, Yingxin Yang, Hui Kong, Yan Zhao, Huihua Qu

**Affiliations:** ^1^ School of Traditional Chinese Medicine, Beijing University of Chinese Medicine, Beijing, China; ^2^ Qingdao Eighth People’s Hospital, Qingdao, Shandong, China; ^3^ School of Chinese Materia Medica, Beijing University of Chinese Medicine, Beijing, China; ^4^ Center of Scientific Experiment, Beijing University of Chinese Medicine, Beijing, China

**Keywords:** carbon dots, Vaccaria Semen, antifibrotic, anti-inflammatory, antioxidant

## Abstract

**Background:** Liver fibrosis represents an intermediate stage in the progression of liver disease, and as of now, there exists no established clinical therapy for effective antifibrotic treatment.

**Purpose:** Our aim is to explore the impact of Carbon dots derived from *Vaccaria Semen* Carbonisata (VSC-CDs) on carbon tetrachloride-induced liver fibrosis in mice.

**Methods:** VSC-CDs were synthesized employing a modified pyrolysis process. Comprehensive characterization was performed utilizing various techniques, including transmission electron microscopy (TEM), multiple spectroscopies, X-ray photoelectron spectroscopy (XPS), and high-performance liquid chromatography (HPLC). A hepatic fibrosis model induced by carbon tetrachloride was utilized to evaluate the anti-hepatic fibrosis effects of VSC-CDs.

**Results:** VSC-CDs, exhibiting a quantum yield (QY) of approximately 2.08%, were nearly spherical with diameters ranging from 1.0 to 5.5 nm. The VSC-CDs prepared in this study featured a negative charge and abundant chemical functional groups. Furthermore, these particles demonstrated outstanding dispersibility in the aqueous phase and high biocompatibility. Moreover, VSC-CDs not only enhanced liver function and alleviated liver damage in pathomorphology but also mitigated the extent of liver fibrosis. Additionally, this study marks the inaugural demonstration of the pronounced activity of VSC-CDs in inhibiting inflammatory reactions, reducing oxidative damage, and modulating the TGF-β/Smad signaling pathway.

**Conclusion:** VSC-CDs exerted significant potential for application in nanodrugs aimed at treating liver fibrosis.

## Graphical Abstract

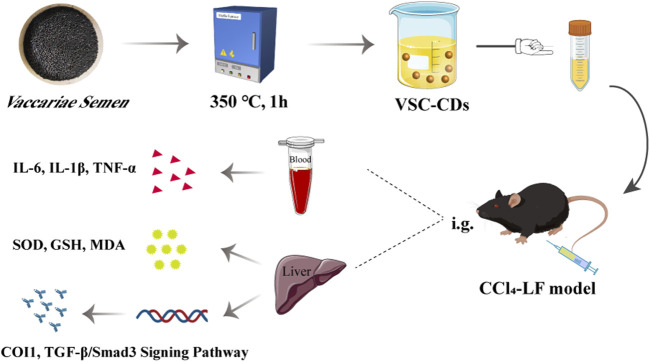

## Introduction

Liver fibrosis (LF), as a wound-healing response, is caused by various chronic persistent liver injury (Cheng et al., 2021; Seitz and Hellerbrand, 2021) including viral hepatitis, alcoholic liver disease (Yang et al., 2021), and non-alcoholic fatty liver disease and has garnered considerable attention (Kumar et al., 2021). Liver fibrosis, including subsequent inflammatory responses and oxidative stress following liver injury, is characterized by the excessive deposition and accumulation of extracellular matrix proteins (Bataller and Brenner, 2005). If it is not promptly prevented and treated, continued progression of liver fibrosis can eventually lead to cirrhosis and even hepatocellular carcinoma. Some research attributes this to the removal or elimination of chronic liver injury resulting from various factors (Huang et al., 2023; Pellicano et al., 2023; You et al., 2023; Zhang et al., 2023) (suppression of extracellular matrix accumulation, anti-inflammatory, and anti-oxidative stress). However, no approved therapy for LF was used in clinical trials, which is in contrast with the robust effects of many anti-LF candidate drugs exhibited in experimental animal models. To date, methods of effective treatment remain unclear except for liver transplant surgery (Deng et al., 2022), which plagues clinical workers and scientific researchers. Therefore, finding innovative pharmacotherapeutic methods to relieve symptoms and arrest the course of liver fibrosis is urgently needed.

Carbon dots (CDs) with ultrafine sizes less than 10 nm are an emerging carbon nanomaterial that has aroused many medical researchers’ interest owing to their diverse physicochemical properties, such as having superior biocompatibility (Belza et al., 2021), photostabilities (Kumar et al., 2022), low cytotoxicitiesties (Mu et al., 2021), and excellent water dispersion (Cai et al., 2021). Additionally, CDs are simple to functionalize due to the multitude of functional groups on their surfaces. Of note, these advantageous properties contributed to the biomedical application of CDs (Liu et al., 2019; Luo et al., 2021; Mansuriya and Altintas, 2021; Wang et al., 2022). Moreover, the development of CDs with inherent bioactivity impelled the progress and innovation of nanotechnology, especially nanomedicine. It is noteworthy that exploration of several medical bioactivities concerning CDs (antitumor (Cai et al., 2021), anti-inflammatory (Hu et al., 2016), anti-oxidant (Wei et al., 2019), hemostasis (Yan et al., 2017)) remarkably exhibited potential strategies for unsolved clinical conundrums like liver fibrosis.

Several studies have focused on exploring the antifibrotic effect of nanodrugs and CDs (Boey et al., 2021; Ren et al., 2021; Xu et al., 2022). Furthermore, the emerging nanotherapeutics could remodel the hepatic fibrotic microenvironment (Zhao et al., 2023), and nanoparticles have served targeted drug delivery of synthetic molecules for management of liver fibrosis based on inflammation and oxidative stress (Singh et al., 2023; Vyas and Patel, 2023). It follows that carbon-based nanoparticles play a crucial role in the treatment of liver diseases. Traditional Chinese medicine (TCM) is economical, shows lower biotoxicity, easily available, and widely used. Our research team have discovered that *Paeoniae Radix Alba carbonisata*-derived carbon dots exhibit a prominent hepatoprotective effect (Zhao et al., 2020). In addition, novel CDs derived from *Junci Medulla Carbonisata* perform hepatoprotective bioactivity (Cheng et al., 2019). Accordingly, functional carbon dots synthesized from green precursors possess unique advantages and extensive potential for exploitation and utilization for LF.

As a traditional Chinese medicine, *Vaccariae Semen* (VS) has a long clinical application history. VS, the dried mature seeds of *Vaccaria Segetalis (Neck) Garcke,* is named after ‘Wangbuliuxing’ in Chinese and recorded in the ancient Chinese classic medical book “the *Synopsis of the Golden Chamber*” by Zhang Zhongjing in the Eastern Han Dynasty: Incinerate VS into ashes without excessive burning. Originally utilized in the treatment of metal knife wounds, through the accumulation of medical experience, processed (fried) VS has been integrated into traditional Chinese medicine formulations for the treatment of liver diseases. We found that in patents related to traditional Chinese medicine formulations for liver diseases (hepatitis, cirrhosis), the use of VS or stir-fried VS is also indicated. Following these clues, we have learned that VS is applied in traditional Chinese medicine formulations for chronic hepatitis B liver fibrosis. From a contemporary pharmacological perspective, certain active ingredients in VS exhibit a variety of properties, such as anti-inflammation (Sun et al., 2017; Gong et al., 2019) and antioxidant effects (Yuan et al., 2014). Therefore, it is reasonable to persist in exploring the therapeutic effects of *Vaccaria Semen* Carbonisatum (VSC), the product of prepared VS on LF. Moreover, we explore the material basis and mechanism of action of VSC.

**FIGURE 1:**
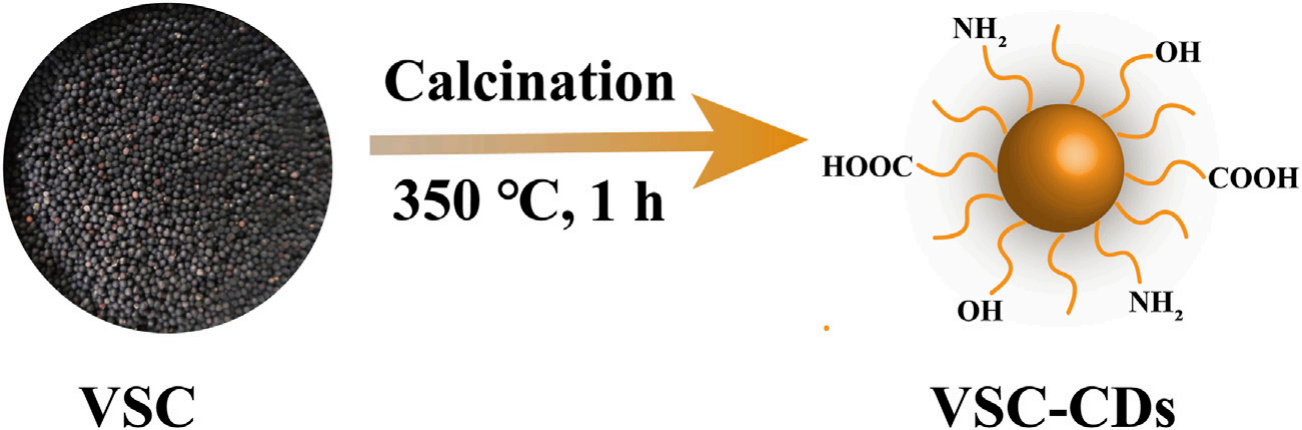
Illustration for as-prepared *Vaccaria Semen* Carbonisatum-based carbon dots (VSC-CDs) by one-step calcination method.

In this study, we identified and synthesized a novel nano-component named *Vaccaria Semen* Carbonisatum-derived Carbon dots (VSC-CDs) utilizing a green and one-step pyrolysis method (Figure 1). Besides, based on identification of their physicochemical characteristics (e.g., morphology, optical properties, functional groups and carried negative charges), we evaluated the alleviation effect of VSC-CDs on LF induced by the carbon tetrachloride (CCl_4_) as well as the cytotoxicity of RAW264.7 cells and blood compatibility. Our study, to the best of our knowledge, focused on inflammation (IL-6, IL-1β, TNF-α), oxidative stress (SOD, GSH, MDA) in the process of CCl_4_-induced LF, and the preliminary mechanism of the alleviation effect of VSC-CDs by detecting the contents of collagen1 (COI1), transforming growth factor-β1 (TGF-β1), Smad3, and α-smooth muscle actin (α-SMA). The results indicate that VSC-CDs exhibit the ability to reduce inflammation levels, enhance antioxidant capacity, facilitate the restoration of impaired liver function in mice, and alleviate liver fibrosis. Our findings hold promise for the development of a novel, environmentally friendly, and effective nanomedicine, offering a potential therapeutic strategy for mitigating liver fibrosis in clinical applications.

## Material and methods

### Chemicals

VS was purchased from Beijing Qiancao Herbal Pieces Co., Ltd. (Beijing, China), and the VSC was prepared in a muffle furnace in our research facility. A dialysis membrane of 1,000 Da molecular weight cut-off was purchased from Beijing Ruida Henghui Technology Development Co., Ltd. (Beijing, China). The Cell Counting Kit-8 (CCK-8) was obtained from Dojindo Molecular Technologies, Inc. (Kumamoto, Japan), and Silybin was purchased from Tianjin Tasly Sants Pharmaceutical Co., Ltd. (Tianjin, China). Carbon Tetrachloride (CCl_4_) was obtained from Beijing InnoChem Science & Technology Co., Ltd. (Beijing, China). Other analytical-grade chemical reagents were acquired from Sinopharm Chemical Reagents Beijing (Beijing, China). Mouse IL-6, IL-1β, and TNF-α enzyme-linked immunosorbent assay (ELISA) kits were brought from Cloud-Clone Crop. (Wuhan, China). SOD, GSH, and MDA kits were from Nanjing Jiancheng Bioengineering Institute (Nanjing, China). Mouse monocyte-macrophage RAW264.7 cells were purchased with Peking Union Cell Bank (Beijing, China). Deionized water was available in all experiments.

### Animals

Adult male C57BL/6J mice (weighing 20.0 ± 2.0 g) were purchased from SiPeiFu Biotechnology Co., Ltd. (Beijing, China) with a Laboratory Animal Certificate of Conformity and raised in a controlled laboratory environment with a well-ventilated room, suitable temperature (24.0°C ± 1.0°C), and relative humidity (45.0%–55.0%) under a 12 h light/dark cycle. The animals were provided with plenty of food and water throughout the experiment. The execution of experimental protocols was strictly in accordance with the Guidelines for Care and Use of Laboratory Animals and approved by the Ethics Review Committee of Animal Experimentation of the Beijing University of Traditional Chinese Medicine.

## Preparation and characterization of VSC-CDs

### Preparation of VSC-CDs

Previously weighed VS dried herbs were placed in a crucible and covered with aluminum foil, which was put into a muffle furnace for high-temperature calcination at 350°C for 1 h (TL0612, Beijing Zhong Ke Aobo Technology Co., Ltd., Beijing, China). The carbonized product, *Vaccaria Semen* Carbonisatum (VSC), was ground into fine powder after it had naturally cooled to 30 °C. Then, 30-fold deionized water (DW) was added to 30 g of VSC powder, and the VSC was boiled twice at 100°C for 1 h each time. The aqueous decoction was subjected to filtration through a 0.22 μm microfiltration membrane, followed by consolidation and concentration of the resulting filtrates utilizing a rotary evaporator. To further remove small molecules and non-carbonaceous impurities, the solution was dialyzed against DW through a dialysis membrane (MWCO = 1,000) for 72 h, which was stored at 4°C for further use.

### Characterization of VSC-CDs

The morphological features and particle size distribution of the VSC-CDs were observed using transmission electron microscopy (TEM; Tecnai G220, FEI Company, Hillsboro, OR, United States). Meanwhile, atomic lattice fringes and structural details were examined by high-resolution transmission electron microscope (HRTEM; JEN-1230, Japan Electron Optics Laboratory, Tokyo, Japan). The fluorescent performances and ultraviolet-visible (UV-vis) absorption spectra of the VSC-CDs were measured via a performing fluorescence spectrophotometer (FL; F-4500, Tokyo, Japan) and an ultraviolet spectrophotometer (CECIL, Cambridge, United Kingdom), respectively. Fourier transform infra-red (FTIR) spectrum (Thermo Fisher, California, United States) data were collected to identify functional groups in VSC-CDs, and the zeta potential values were calculated using a malvern zetasizer nano ZS90 (Malvern Instruments, United Kingdom). X-ray photoelectron spectroscopy (XPS; ESCALAB 250Xi, Thermo Fisher Scientific, Fremont, CA) was used to record the surface composition and chemical elements of VSC-CDs with a mono X-ray source Al Kα excitation (1,486.6 eV).

### Quantum yield of VSC-CDs

We chose Quinine sulfate (quantum yield [QY]: 54%, 0.1 M sulfuric acid [H_2_SO_4_]) as the reference sample to measure the comparative QY of VSC-CDs. To minimize the reabsorption effect, the absorbance of the CDs and the R were kept under 0.05. QY of VSC-CDs was calculated by the following Formula 1:

$${\text{QY}}_{\text{CDs}}={\text{QY}}_{R}\;✕\;\frac{I_{\text{CDs}}}{I_{R}}\times \frac{A_{R}}{A_{CDs}}\times \frac{\eta_{CDs}^{2}}{\eta_{R}^{2}}$$ (1)

where QY is fluorescence quantum yield, *I* represent an integrated area of emission intensity, and *A* and *ƞ* indicate the absorption value at 371 nm wavelength and the refractive index of the solvent, respectively. The CDs and the R denote VSC-CDs and the reference sample, respectively.

### Fingerprint analysis of VSC and VSC-CDs by high-performance liquid chromatography

The individual components determination of VSC and VSC-CDs was detected by HPLC (Agilent LC-1260, Waldbronn, Germany) with a C-18 column (250 mm × 4.6 mm × 0.5 μm, ZORBAX SB-C18, United States). The mobile phase consisted of methanol (A) and 0.3% phosphoric acid (B). The gradient elution procedure was as follows: 35%–35% A at 0–3 min; 35%–40% A at 3–5 min; 40%–45% A at 5–10 min; 45%–40% A at 10–20 min. The flow rate of the mobile phase was 1 mL/min, the column temperature was 30°C, the detection wavelength was 254 nm, and the injection volume was 5 µL. Furthermore, all samples were purified by filtration through a 0.22 μm cellulose membrane and analyzed in triplicate.

## *In vitro* assay

### Cytotoxicity and hemocompatibility assay of VSC-CDs

RAW 264.7 cells were used to measure the cytotoxicity of VSC-CDs using a CCK-8 assay *in vitro*. Firstly, RAW 264.7 cells were cultured in Dulbecco’s modified Eagle’s medium (DMEM) supplemented with 20% fetal bovine serum at 37°C in a humidified 5% CO_2_. Then the cells were seeded in a 96-well plate at a density of 1 × 10^4^ cells per well and incubated for 24 h. After disposing of the original medium in each well, different concentrations of VSC-CDs were added (1,000, 500, 250, 125, 62.5, 31.25, 15.62, and 7.81 μg/mL) to the designated wells. Another 24 h later, the medium containing VSC-CDs was discarded and the cells were washed with phosphate-buffered saline (PBS) twice. Subsequently, we added 10 μL CCK-8 solution and incubated for 4 h. The absorbance of each well was detected by a microplate reader (Bitoke, VT, USA) at a wavelength of 450 nm, and cell viability was calculated according to the following Formula 2:

$$\text{Cell Viability}\left(\%\text{ of control}\right)=\frac{A_{e}‐A_{b}}{A_{c}‐A_{b}}\times 100$$ (2)

Ae, Ab, and Ac represent the experimental, blank, and control groups, respectively, at 450 nm.

Hemolysis assay of VSC-CDs was carried out according to the method described by previous report. The red blood cells (RBCs) were centrifugated at 3,500 rpm for 15 min in a constant temperature centrifuge at 4°C and were washed three times with PBS (pH = 7.2–7.5). The sample was added into a 2 mL EP tube with 500 μL 10% RBC suspension and mixed with different concentration VSC-CDs solution (1,000, 800, 400, 200, and 100 μg/mL) in the same volume at room temperature. Subsequently, this sample was incubated at 37°C for 2 h and 4 h. The hemolysis ratio was calculated according to the following Formula 3:

$$\text{Hemolysis}\left(\%\right)=\frac{A_{\text{Sample}}‐A_{\text{PBS}}}{A_{\text{Water}}‐A_{\text{PBS}}}\times 100$$ (3)

When the hemolysis ratio was lower than the internationally recognized standard (5%), we believed that the medicine had high blood compatibility.

## *In vivo* experiments

### Models of hepatic fibrosis and drug treatment

Male C57BL/6J mice were randomly allocated into six groups (*n* = 7) as follows: control group (normal saline [NS] 10 mL/kg, p.o.), model group (CCl_4_ 10 mL/kg, p.o.), positive group (silybin 10 mL/kg, p.o.), and the high-, medium-, and low-dose VSC-CDs groups (3.63, 1.81, and 0.91 mg/kg, respectively, intraperitoneally). All groups were injected intraperitoneally with the prepared carbon tetrachloride oil solution (10 mL/kg) for 8 weeks, two times per week, with a 3-day interval, apart from the control group, where mice were given an intraperitoneal injection of an equal volume of corn oil. Then, we performed oral administration for mice in the VSC-CDs groups. The mice of the control group and the model group were given an equivalent volume to normal saline positive group. The mice of the positive group were given silybin. Body weight was recorded daily at a regular time until the end of the experiment to count and analyze the weight difference. A total of 12 h after the final administration, all mice were sacrificed. The liver was isolated and weighted to gain the weight ratio of liver to body (liver/body wt%) and photographed for observing morphological changes.

### Histopathological observation

After removing liver tissue rapidly and rinsing it with ice-cold saline, a small piece of liver tissue was cut and fixed in 4% paraformaldehyde, dehydrated, and embedded in paraffin. Then 4 μm thick sections were prepared for H&E, Masson staining, and Sirius red staining. Morphological changes were observed with an optical microscope to compare the severity of liver injuries and the fibrosis status among various treatment groups.

### Detection of biochemical indexs

All mice were anaesthetized with 4% chloral hydrate (0.40 g/kg), and retro-orbital blood samples were collected into an Eppendorf tube to clot overnight at 4°C, centrifuged at 750 × g for 15 min, and then the blood serums were separated from whole blood. One part of the sera samples was determined to analyze the changes in alanine transaminase (ALT) and aspartate aminotransferase (AST) activity and the levels of total bile acid (TBA), total bilirubin (TBIL), and cholesterol (TC) by an automatic biochemical analyzer (XI-800, Sismecon Co., Ltd, Japan). Other part of those sera samples was used to examine the contents of tumor necrosis factor-α (TNF-α), interleukin (IL)- 6, and interleukin (IL)- 1β in serum detected using corresponding kits (Nanjing Jiancheng Biochemical Reagent Co., Nanjing, China) according to the manufacturer’s instructions. What is more, after taking out the liver tissues from the different groups stored at −80°C, those tissue samples were cut into small pieces, homogenized with PBS on ice, and then centrifuged at 750 × g for 15 min. The gained supernatants were collected to determine levels of SOD, GSH, and MDA using respective kits.

### Western blot analysis

Total protein extracted from liver tissue in a RIPA buffer (Beyotime Institute of Biotechnology, Shanghai, China) was used for Western blot analysis. The BCA Protein Assay Kit was applied to quantitate protein content following the manufacturer’s protocol. Proteins were separated by gel electrophoresis and transferred to membranes, followed by blocking in 1× TBST (5% *w*/*v* skim milk) for 2 h at room temperature and probing overnight with primary antibodies against COI1 (1:3000), TGF-β1 (1:4000), Smad3 (1:4000), α-SMA (1:5000), and GAPDH (1:8000) at 4°C. After washing membranes and incubating with horseradish peroxidase-conjugated secondary antibodies (1:8000) for 1 h, the films were scanned densitometrically. By considering the GAPDH density as an internal control, the gray densities of the protein bands were normalized and quantified using ImageJ software.

### Statistical analysis

All statistical analysis was carried out and analyzed using IBM SPSS statistics software, version 20.0. data, with normal distribution, and uniform variance were shown as means ± standard deviation (SD). One-way analysis of variance (ANOVA) was performed to compare statistically significant differences with the least significant difference (LSD) test used for multiple comparisons. Data with non-normal distribution non-normally distributed data were analyzed using non-parametric statistics using the Kruskal–Wallis test and a *post hoc* test; *p* < 0.05 and *p* < 0.01 were taken as statistically significant differences.

## Results

### Characterization of VSC-CDs

**FIGURE 2:**
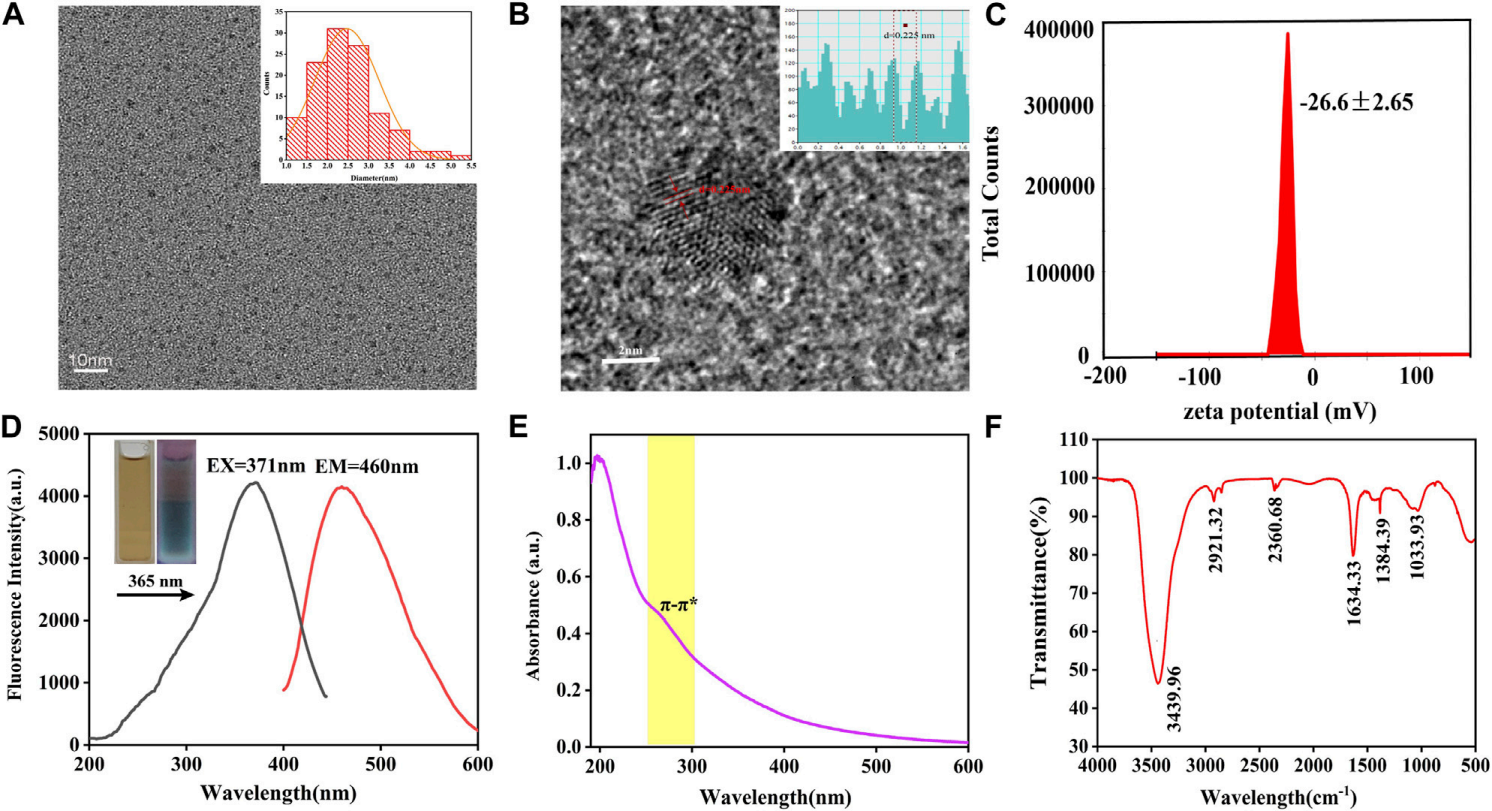
TEM images of VSC-CDs and histogram depicting the **(A)** particle size distribution, **(B)** HR-TEM image, **(C)** zeta potential, **(D)** excitation and emission spectrum, **(E)** ultraviolet-visible spectrum, and **(F)** FTIR spectrum.

The TEM image showed the morphology and size distribution of as-prepared VSC-CDs. As depicted in Figure 2, the CDs were almost spherical with favorable homogeneous dispersibility and the particle size distribution of VSC-CDs ranged primarily from 1.0–5.5 nm based on factual examination and calculation of hundred particles (Figure 2A). The HRTEM image (Figure 2B) revealed that the distinct lattice spacing was 0.23 nm. Furthermore, the zeta potential of VSC-CDs dissolved in aqueous solution was −26.6 ± 2.65 mV (Figure 2C), indicating VSC-CDs attained a state of moderate stability. The surface of VCS-CDs contains numerous oxygen-containing functional groups, making it susceptible to releasing hydrogen ions and rendering the solution negatively charged. Comparatively, carbon dots with negative charge exhibit lower toxicity (Havrdova et al., 2016; Yan et al., 2018), and the negative potential of nanoparticles contributes to their *in vivo* dispersibility and stability (Tang et al., 2023) and diminishes aggregation of the particles in the solution (Hou et al., 2023).

The spectral properties were exhibited next. The fluorescence emission spectra demonstrated a maximum emission at 460 nm and maximum excitation at 371 nm in the emission and excitation spectrum (Figure 2D). The aqueous solution of VSC-CDs was brown in the room light, giving off blue fluorescence under 365 nm ultraviolet lamp. The UV-Vis absorption spectra of VSC-CDs indicated a weak absorption peak at 260 nm, which was attributed to the conjugated C=C bonds π–π* electron transition of VSC-CDs (Figure 2E). Meanwhile, the QY of VSC-CDs was calculated to be 2.08% using quinine sulphate as a reference. The aforementioned results indicate that VSC-CDs manifest photoluminescent properties.

In terms of surface functional groups and chemical characteristics of the VSC-CDs, FTIR spectra (Figure 2F) displayed a strong characteristic peak at 3,439 cm^−1^ attributed to the absorption bands of O–H. The absorption peak located at 2,921 cm^−1^ was assigned to -C-H stretching vibration, suggesting the existence of -CH_3_ and -CH_2_ groups in VSC-CDs. Moreover, the peak at 2,360 cm^−1^ showed the presence of the -C≡N bond. The peak observed at 1,634 cm^−1^ was associated with -C=O stretching vibration. Both peaks appearing at 1,384 cm^−1^ and 1,033 cm^−1^ were corresponding to -C-N and -C-O-C bonds separately. The above-mentioned results indicate that the surface of VSC-CDs features multifunctional groups, including carbonyl, carboxyl, and hydroxyl moieties, those organic functional groups enhanced water-solubility on VSC-CDs.

**FIGURE 3:**
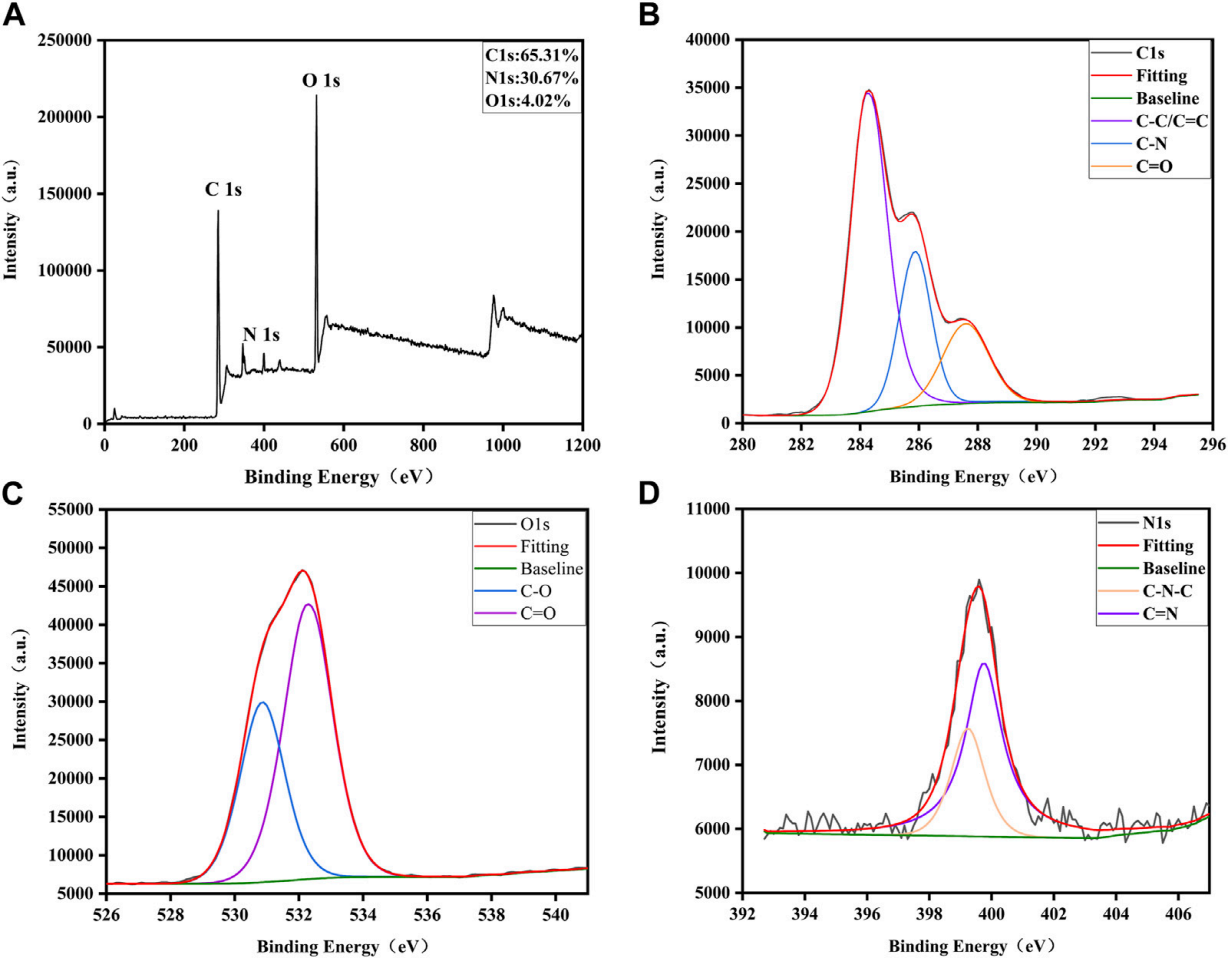
Surface composition and elemental analysis of VSC-CDs by XPS. **(A)** XPS spectra of VSC-CDs, **(B)** C 1s, **(C)** O 1s, and **(D)** N 1s.

XPS further disclosed the element composition and allocation information of VSC-CDs (Zhao et al., 2021). As shown in Figure 3A, three peaks were evident at 284.94, 399.67, and 531.8 eV, which indicated the purified CDs were mainly composed of C (65.31%), O (30.67%), and a small amount of N (4.02%). The C 1s spectrum (Figure 3B) deconvoluted into three peaks at 284.28, 285.85, and 287.61 eV and was related to the C-C/C=C, C-N, C=O groups (Han et al., 2019; Wang et al., 2020; Zhang et al., 2020). In the high-resolution O 1s spectrum (Figure 3C), the O 1s peak consisted of two subpeaks at 530.83 eV (C-O) (Zhang et al., 2020) and 532.28 eV (C=O) (Wang et al., 2020). Two peaks at 399.24 and 399.75 eV were presented in the high-resolution N 1s spectrum (Figure 3D), representing the presence of C-N-C and C=N bonds. The XPS spectra substantiated the presence of diverse functional groups, encompassing carbonyl, carboxyl, and hydroxyl on the surface of VSC-CDs. All the above results were in line with FTIR analysis.

**FIGURE 4:**
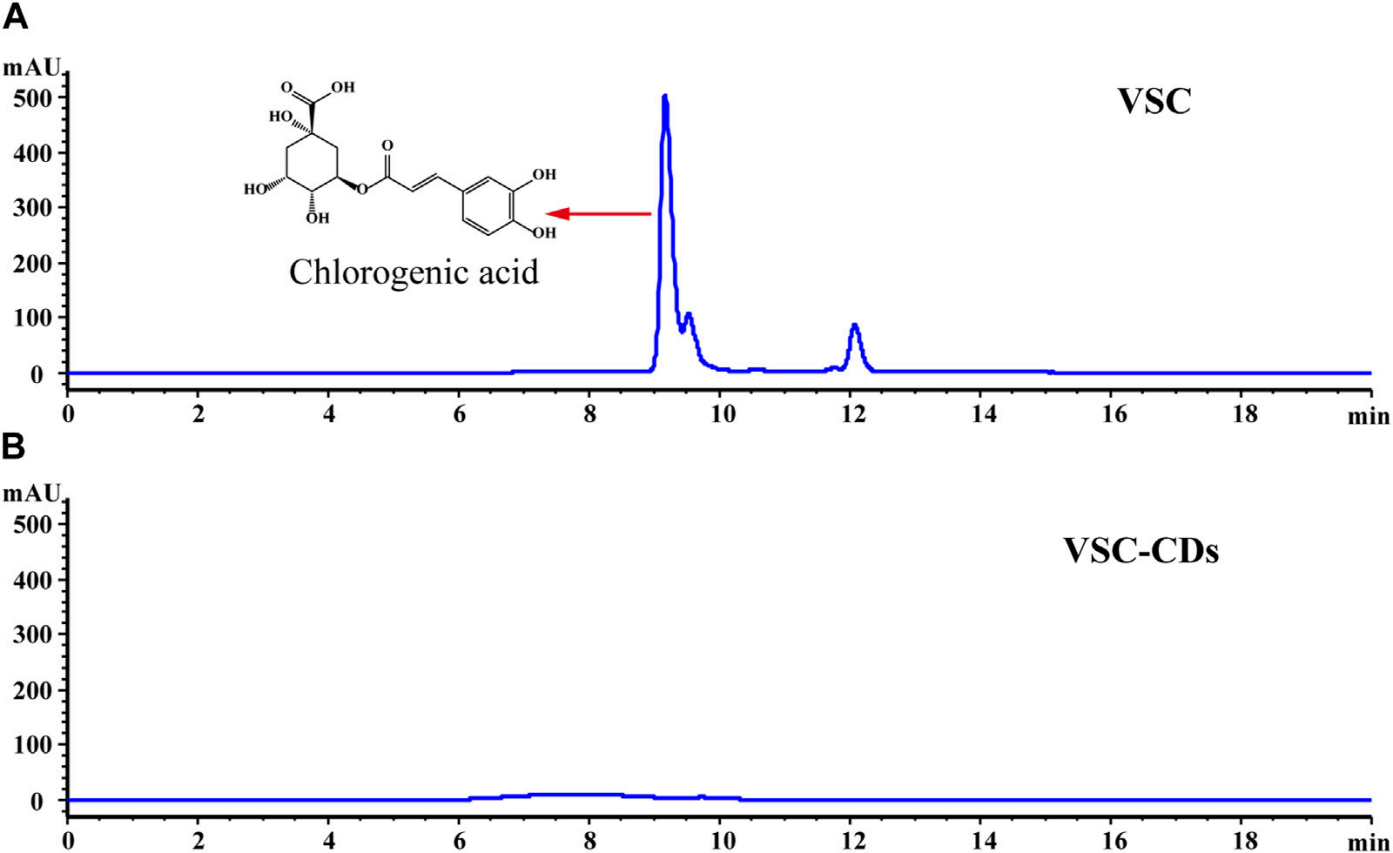
High-performance liquid chromatogram of **(A)** VSC and **(B)** VSC-CDs.

The high-performance liquid chromatogram results of the VSC and VSC-CDs are shown in Figure 4. Chlorogenic acid is the main small molecule in VSC, while the corresponding characteristic peaks disappeared in VSC-CDs, which indicated no active small-molecule compounds in VSC-CDs.

### Cytotoxicity evaluation and hemocompatibility

**FIGURE 5:**
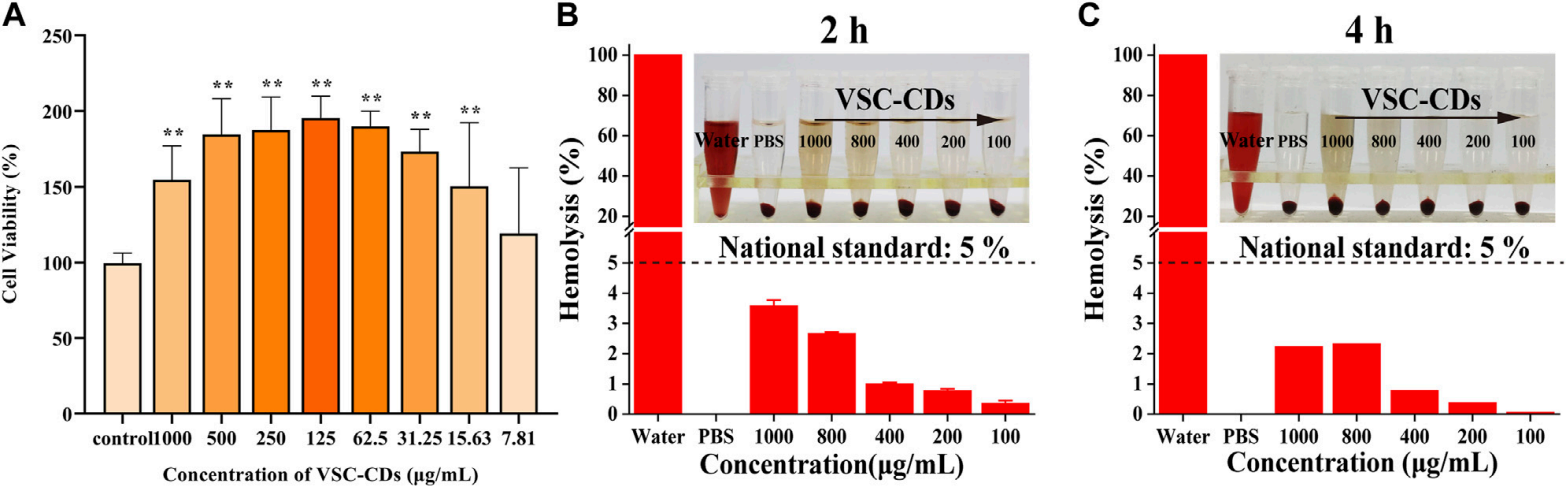
**(A)** Effect of different concentrations of VSC-CDs on cell viability of the RAW264.7 macrophage; **(B,C)** hemolytic assay of VSC-CDs for 2 h and 4 h.

To investigate the cytotoxicity of VSC-CDs, a CCK-8 assay was carried out. RAW264.7 cells were cultured to different concentrations of VSC-CDs (7.81–1,000 μg/mL) for 24 h. Figure 5A illustrated that the viability of RAW264.7 cells treated with VSC-CDs was all over 100% compared with the control cells. Cell viability increased steadily between 7.81 μg/mL and 125 μg/mL, reaching as high as 195% when the concentration of VSC-CDs was up to 125 μg/mL. The proliferation effect gradually got weaken at values of ranging from 125 to 1,000 μg/mL. In conclusion, VSC-CDs possessed scarce biotoxicity and good biocompatibility, which means VSC-CDs have a good application prospect for drugs.

Hemolysis assay is typically used in biocompatibility testing to evaluate the blood safety of materials. Intuitively, as Figures 5B,C show, all doses of VSC-CDs did not cause significant hemolysis of red blood cells. Moreover, this property of VSC-CDs does not change as the incubation time changes (for 2 h and 4 h). Accordingly, VSC-CDs are highly hemocompatible.

## The VSC-CDs mitigated CCl_4_-induced LF model

### Effect of VSC-CDs on body weight and liver/body wt%

**FIGURE 6:**
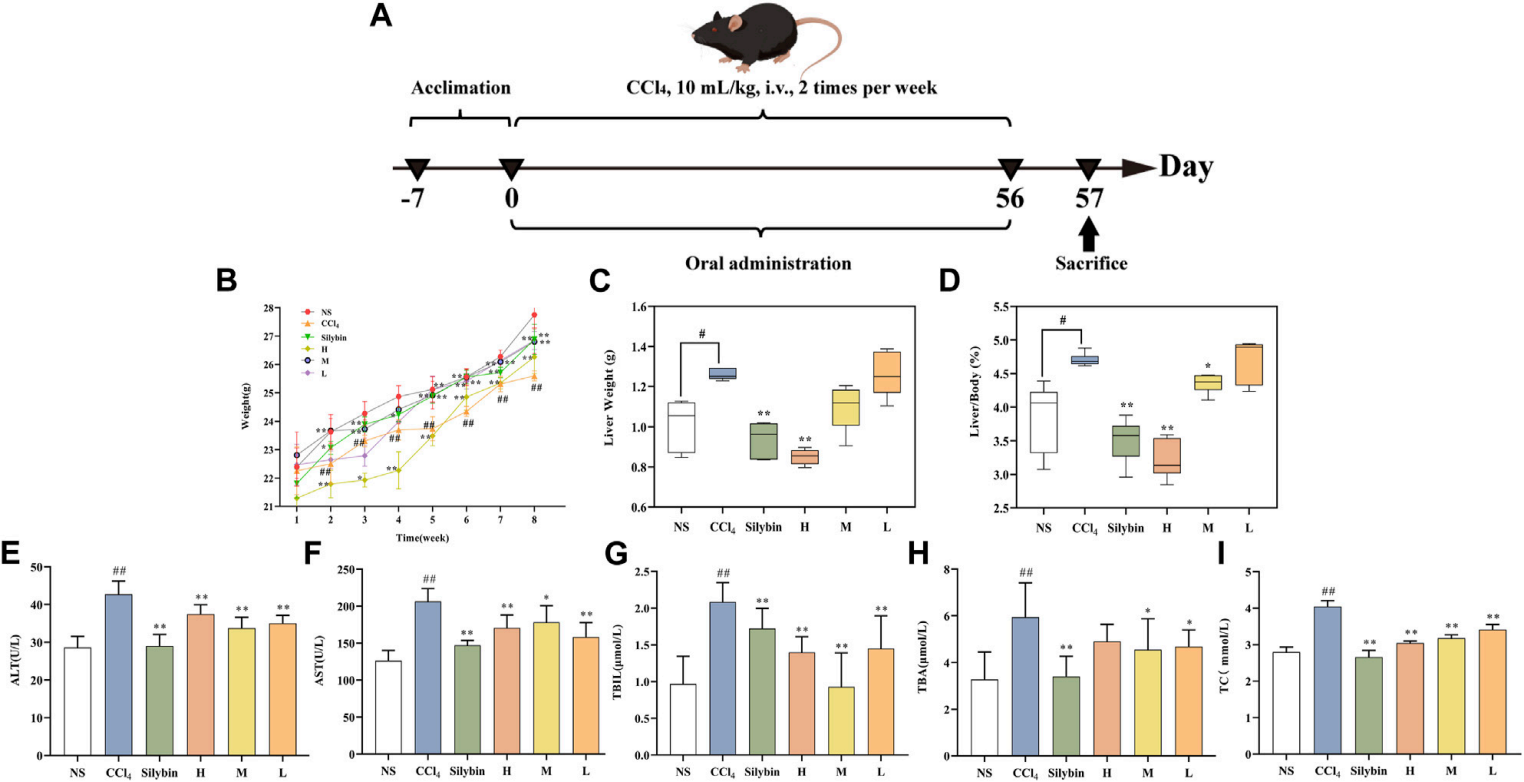
**(A)** schemes of RM-AKI model **(B–D)** the effect of VSC-CD_S_ on the body weight, liver weight, and liver index of mice with liver fibrosis **(E–I)** biomarker measurements including ALT, AST, TBIL, TBA, and TC vs. control group #*p* < 0.05 and ##*p* < 0.01, vs. Model group *p < 0.05 and **p < 0.01. **(H)** High dose; L: Low dose; M: Medium dose; NS: Normal saline.

In Figure 6A, after almost 2 months of molding and drug administration, the mice were then sacrificed. We recorded the weight every week (Figure 6B); in the second week, the weight profile showed that in contrast to the control group, body weight of the CCl_4_-induced group mice was decreased (*p* < 0.01), while VSC-CDs of the high- and medium-dose groups remarkably counteracted (*p* < 0.01) weight loss in mice. In the next week, the body weight of the positive group was also significantly elevated (*p* < 0.01). Similar observations were made about the mice in the high-, medium-, and low-dose groups treated with VSC-CDs as well as the silybin group for 5–8 weeks. Moreover, the control group displayed a tendency for weight gain throughout the experiment. These results manifested that VSC-CDs could reduce the loss of the body weight by CCl_4_ injection.

The severity of liver disease may be partially reflected by the liver/body weight ratio (Figures 6C,D). To investigate the effects of prolonged exposure to CCl_4_ on organ weight, the weights of the liver samples were assessed and used to determine the liver/body weight ratio. Liver/body wt% and liver weight in the model group were significantly higher (*p* < 0.05) compared with the control group. After the consequent application of VSC-CDs for 8 weeks, liver/body wt% and weight showed a prominent decrease in high-dose (*p* < 0.01) and medium-dose groups (*p* < 0.05) of VSC-CDs. The decreasing trend of liver weight existed also in high-dose of VSC-CDs (*p* < 0.01).

### VSC-CDs inhibited expression of biochemical parameters

As shown in Figures 6E–I, several biochemical markers (ALT, AST, TBIL, TBA, TC) were determined to evaluate hepatocyte injury in serum levels. Figure 6 showed the content of ALT, AST, TBIL, TBA, and TC in the model group were significantly higher than the normal saline group (*p* < 0.01). In contrast with the model group, the serum ALT, TBIL, and TC activity of mice was significantly decreased in the positive group and high-, medium-, and low-dose groups of VSC-CDs (*p* < 0.01). As for the serum content of AST, the model group was higher than the control group, and the medium-dose of VSC-CDs significantly downregulated its levels (*p* < 0.05) compared with the model group. More specifically, the serum content of AST (*p* < 0.01) was substantially suppressed by high and low doses of VSC-CDs and silybin treatment. The medium- and low-dose groups (in both groups: *p* < 0.05) of VSC-CDs exhibited a pronounced reduction as compared to the model group, showing similar results for TBA levels. The results of liver function indicator testing suggest that VSC-CDs can mitigate liver damage induced by CCl_4_.

### VSC-CDs ameliorated CCl_4_-induced histopathology and morphology

**FIGURE 7:**
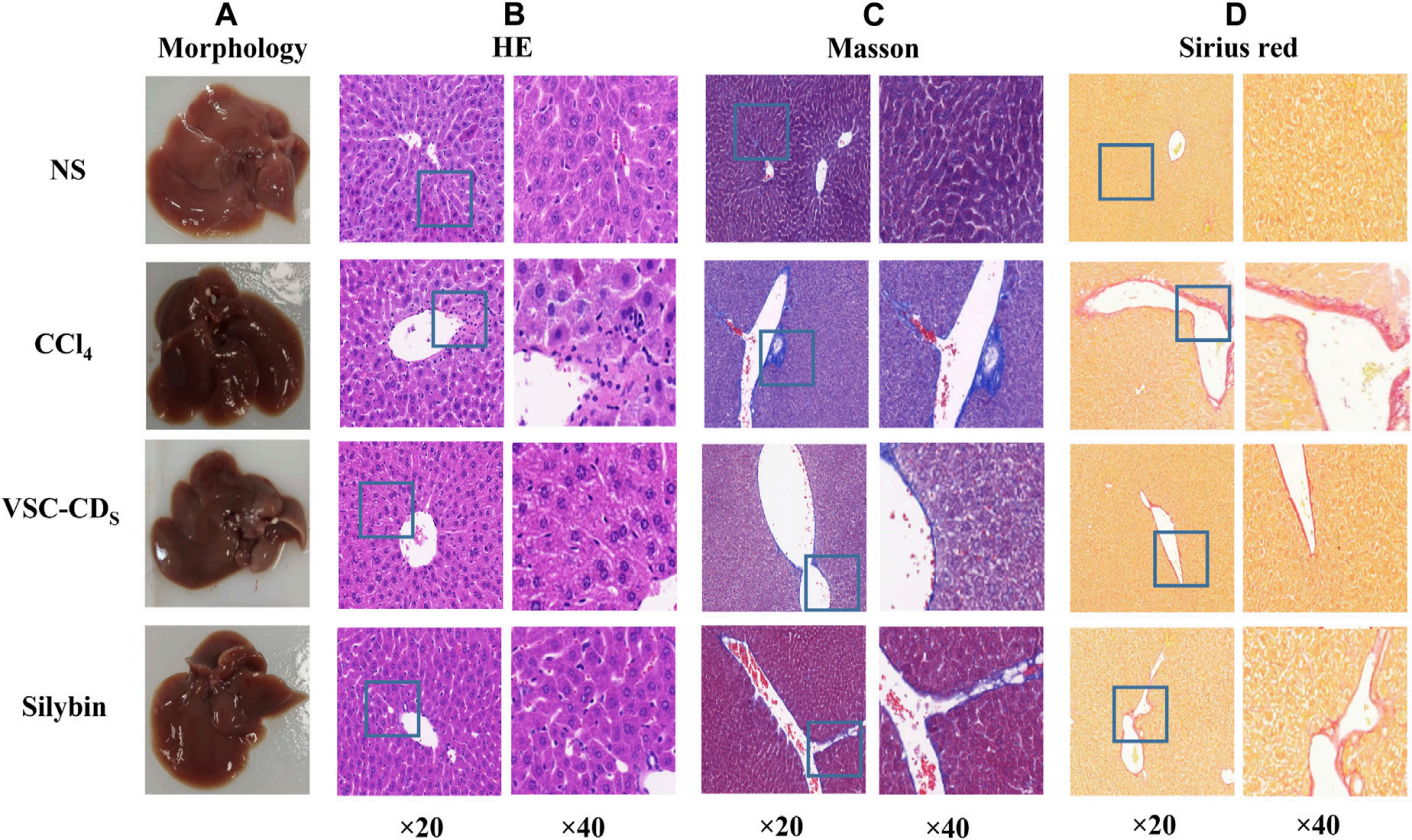
Effects of VSC-CDs on the appearance and pathological changes of liver tissue in CCl_4_-induced liver fibrosis model mice. **(A)** the appearance of liver tissue, **(B)** H&E staining, **(C)** Masson staining, and **(D)** Sirius red staining.

CCl_4_ administration for 8 weeks triggered morphological changes of liver tissue, as evidenced by the roughened surface attached to a granular substance, dull luster, and edge passivation (Figure 7A). Nonetheless, treatment with VSC-CDs mitigated the pathological alterations in liver. Along with the measurement of morphological observation, we performed a histopathological examination of liver tissues. Liver sections of the control group (Figure 7B) stained with H&E showed normal hepatic architecture; liver cells were arranged in plates or cords and radiated from the central venue regions without hepatic steatosis or necrosis. It was worth noting that the liver structure was destroyed and the boundary blurred, accompanied by massive necrosis of liver cells, and infiltration of inflammatory, diffuse microvesicular steatosis lesions by the injection of CCl_4_. In contrast, VSC-CDs treatment significantly attenuated the liver injury and lesions as mentioned in the CCl_4_-administrated group. In addition, Masson staining (Figure 7C) and Sirius red staining (Figure 7D) were employed to detect collagen deposition. We could distinctly observe excessive accumulation and fibrotic septa in the CCl_4_-treated fibrotic liver, while CCl_4_-induced elevation evidently was alleviated by VSC-CDs treatment. Taken together, the observed alterations in pathological slices directly demonstrate that VSC-CDs can inhibit CCl_4_-induced liver fibrosis.

### Effect of VSC-CDs on inflammatory cytokines in serum

**FIGURE 8:**
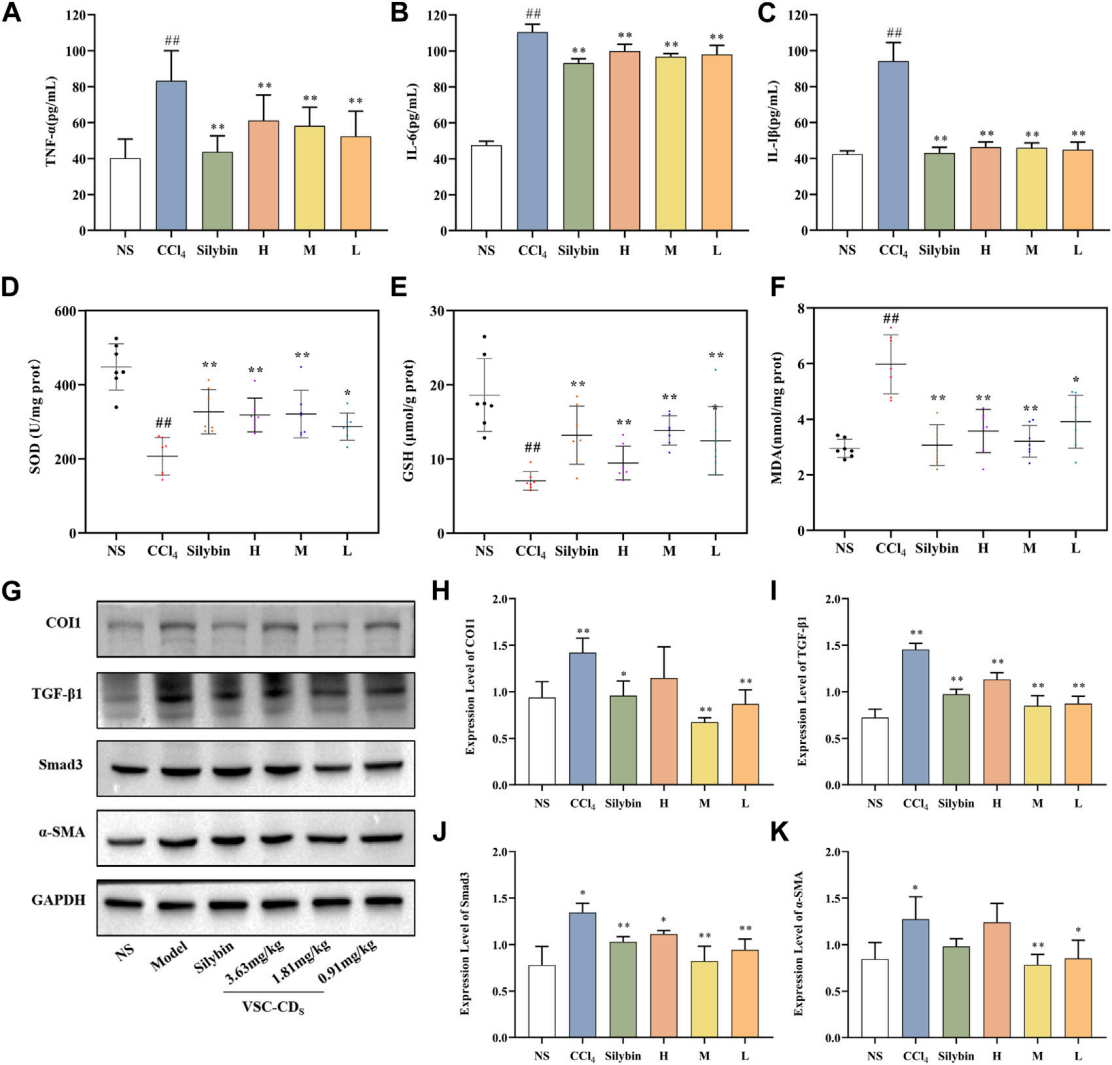
Effects of inflammatory cytokines, antioxidant levels and the expression of TGF-β/Smad signaling pathway-related proteins in the liver tissue of CCl_4_-induced LF model mice. Content of **(A)** TNF-α, **(B)** IL-6, and **(C)** IL-1β; Effects of **(D)** SOD, **(E)** GSH, and **(F)** MDA; the western blot image **(G)** and the expression level of **(H)** COI1, **(I)** TGF-β1, **(J)** Smad3, and **(K)** α-SMA.H: High dose (3.63 mg/mg); M: Medium dose (1.81 mg/kg); L: Low dose (0.91 mg/kg); NS: Normal saline.

Inflammatory mediators play a crucial role in the induction, initiation, progression, and aggravation of liver fibrosis (Zhang et al., 2016a). The levels of TNF-α, IL-6, and IL-1β in liver tissue were determined to determine the anti-inflammatory effect of VSC-CDs. As Figures 8A–C show, in comparison to that in the control group, serum concentrations of TNF-α were dramatically upregulated in the model group (*p* < 0.01). In sharp contrast, silybin and VSC-CDs (high-, medium-, low-dose) inhibited CCl_4_-induced elevated content of TNF-α relative to the model group (*p* < 0.01). The contents of IL-6 and IL-1β were similar to those for TNF-α. Based on the results, VSC-CDs in various dosage groups significantly diminish the levels of TNF-α, IL-6, and IL-1β, signifying that VSC-CDs inhibit the inflammatory response and thereby alleviate liver fibrosis.

### Effect of VSC-CDs on antioxidant levels

Oxidative stress is one of the underlying mechanisms in CCl_4_-induced liver fibrosis; reactive oxygen species can trigger the initiation of the inflammatory cascade to further accelerate the progression of liver fibrosis (Xu et al., 2022). Antioxidant markers (SOD, GSH) and peroxidation product (MDA) (Figures 8D–F) were measured to evaluate the alterations of the redox status.

When it comes to SOD in liver tissue, a significant difference (*p* < 0.01) was seen: the activity of SOD in the model group was visibly restrained compared with the control group. More remarkably, VSC-CDs treatment improved the levels of SOD to a large degree. Otherwise, injection of CCl_4_ resulted in a significant decrease in the activities of GSH compared to the control group, and, as expected, silybin and the different dosages of VSC-CDs also accomplished elevation of GSH levels (*p* < 0.01).

On the contrary, MDA, one of lipid metabolites, must be scavenged in a timely manner to protect hepatocytes from oxidative damage. The assay results indicated that, owing to the intervention of CCl_4_, the contents of MDA inevitably were increased in comparison to in the control group (*p* < 0.01). Of note, the increase was evidently attenuated by VSC-CDs and silybin treatment (*p* < 0.01). Collectively, VSC-CDs could improve the antioxidant capacity of the liver and scavenge oxygen-free radicals to balance the redox system.

According to the results, under the intervention of VSC-CDs, oxidative stress levels decrease, contributing to the mitigation of liver fibrosis along with a reduction in the inflammatory response.

### Effect of related proteins on TGF-β/Smad signaling pathway

The TGF-β/Smad signaling pathway is one of representative signaling pathways in liver fibrosis. We conducted a preliminary investigation on TGF-β/Smad signaling pathway to elucidate tentatively the potential mechanism of action. The results of semi-quantitative analysis (Figures 8G–K) showed the expressions of the COI1 and TGF-β1 proteins was highly elevated compared with the control group (*p* < 0.01), the three VSC-CDs doses (H, M and L: *p* < 0.01) significantly inhibited the levels of TGF-β1, while the expressions of COI1 was notably lessened by M and L-doses (*p* < 0.01) VSC-CD_S_. In addition, the concentrations of Smad3 proteins markedly reduced in silybin (*p <* 0.05) and VSC-CDs groups (H: *p <* 0.05, M and L: *p* < 0.01). α-SMA is considered a marker of Hepatic stellate cell (HSC) activation with CCl_4_ -stimulation, and the expression levels of α-SMA in the model group (*p <* 0.05) was increased distinctively, which indicated CCl_4_ induced the activation of HSCs. Inhibition of the expression of α-SMA was found in VSC-CDs treated groups (M: *p <* 0.01, L: *p <* 0.05) compared to the model group. These findings suggest that the favorable effects of VSC-CDs on the improvement of liver fibrosis may be linked to the modulation of the TGF-β/Smad signaling pathway.

## Discussion

CDs seen as a newly carbon nanomaterial joined latest the carbon family, which gained tremendous concern and research by virtue of its unique biological properties, such as excellent absorbance and optical photoluminescence. Noteworthy, uptake, biodistribution, and excretion of nanoparticles (NPs) are closely linked to many organs, especially the liver. Most of the NPs are taken up by non-parenchymal cells of the liver, then phagocytosed, and delivered to the lysosome by kupffer cells for enzymatic digestion (Zhang et al., 2016b; Cornu et al., 2020; Truskewycz et al., 2022). Meanwhile, physicochemical properties including charge, size, surface functionality, zeta potential and chemical composition, can largely influence the uptake, biodistribution, excretion, and even the toxicity of NPs. As previously reported, hepatocyte uptake increases for NPs with positive zeta potential, contrary to macrophages that preferentially take up negatively charged NPs (Wang et al., 2015). On the excretion of NPs, the particles with positive charge were strongly through the kidney, in contrast, the negatively charged through the liver (Liang et al., 2016). And renal excretion is prevented when the NPs size are above 6 nm (Dogra et al., 2018). Thus, physicochemical properties of nanoparticle were merited to be identificated and characterized to explore their bioactivity.

In recent years, renewable sources were used for the preparation of CDs, including apple (Mehta et al., 2015), oolong tea (Shi et al., 2017), guava leaf (Ramanarayanan and Swaminathan, 2020) as well as natural source of carbon dots from part of the plant and its applications (Humaera et al., 2021), which encouraged and enlightened the developments of CDs choosing biomass as the novel precursors, like charcoal drugs. By using low-cost, low energy consumption, simple manipulation, and one-step preparation method as selection of preparation conditions, VSC-CDs were synthesized with high temperature carbonization. Then VSC-CDs were characterized and identified by a range of characterization instruments. Meanwhile, we have proved that VSC-CDs had hepatoprotective effect on the mice induced by alcohol, which laid the groundwork for this study (Zhao et al., 2021).

Current researches mostly put eyes on nanoparticles as delivery carriers for liver fibrosis therapy (Wang et al., 2021; Xu et al., 2022), whereas the application of CDs for treating liver fibrosis is in its infancy. In this study, novel carbon dots with tiny size (1.0–5.5 nm), zeta potential (−26.6 ± 2.65 mV), chemical composition (-C≡N, -C=O, C-O-C) and optical properties were developed from *Vaccaria Semen* carbonisata. Based on those, we evaluated the alleviation effect of VSC-CDs by using CCl_4_-induced liver fibrosis model in mice and preliminarily explored the underlying mechanism.

Safety is a primary and principal issue in the development of new drugs along of clinical application. In our study, we first assessed the cytotoxicity of VSC-CDs to prepare for the study of follow-up pharmacodynamic effects. VSC-CDs showed no cytotoxicity and exhibited an excellent cell proliferation of RAW264.7 cells, while superior cytocompatibility makes VSC-CDs potential alleviation candidate drug. Hemolysis assay evaluated the blood safety of materials. VSC-CDs did not cause significant haemolysis of red blood cells. Moreover, this high hemocompatiblity of VSC-CDs does not change from 2 h to 4 h), VSC-CDs have excellent blood safety. Undoubtedly, VSC-CDs exhibit high cellular compatibility and excellent blood compatibility. This advantage serves as a prerequisite for their translation into clinical applications.

Herein, we found VSC-CDs extenuated liver injury in LF mice both in macrography and micrography. After administration for 8 consecutive weeks, VSC-CDs exhibited greater efficacy in improving weight and reducing the liver/body ratio compared to treatment with CCl_4_ alone. Concurrently, liver morphology changed positively, and the destruction of hepatic cells was partly restored upon morphological and pathological observations, which might be due to VSC-CDs. To the best of our knowledge, LF is characterized by massive accumulation involved in collagens and additional ECM protein (Dinarello, 2006). Masson staining and Sirius red stained sections illustrated the area covered by collagen deposition was lessened. These results definitely attested to impairment of liver deformity and anti-fibrotic activity which matched the previous study (Khalil et al., 2021). The observed alterations in the appearance of liver tissues and pathological slices constitute evidence confirming that VSC-CDs can alleviate CCl_4_-induced liver damage and mitigate liver fibrosis.

The liver was harmed by multiple pathogenic agents, which led to hepatic fibrosis. It is well-documented that an inflammatory micro-environment, oxidative stress, and excessive deposition of ECM must be orchestrated to inhibit the progress of liver fibrosis (Liu et al., 2022; Rodríguez et al., 2021). Many other studies confirmed CCl_4_ induced a liver fibrosis model (liver cirrhosis), which has applied extensively by a multitude of researchers (Scholten et al., 2015; Cong et al., 2017). In addition, liver function problems, inflammation, and oxidative stress induced by CCl_4_ have been identified as closely associated with liver fibrosis. In our investigations, we conducted research specifically addressing these aspects.

Serum parameters of liver function (ALT, AST, TBA, TBIL, TC) were detected. The liver enzymes (ALT, AST) and biliary index (TBA, TBIL, TC) in serum are markers of liver inflammation and necrosis in liver tissue (Mohamed et al., 2015). ALT and AST levels of animals exposed to CCl_4_ in serum were significantly improved, which is in agreement with previous studies (Khalil et al., 2021). In this study, treatment with VSC-CDs downregulated the increment of ALT and AST. The metabolism of TBA and TBIL is determined by liver function (Zhao et al., 2020). CCl_4_ injection increased the contents of TBA and TBIL in blood serum, which indicated liver function was damaged. Besides, the serum levels of TC were also increased due to abnormal lipid metabolism caused by CCl_4_. Our results showed that VSC-CDs suppressed the release of TBA, TBIL, and TC and diminished liver damage by impairing liver function. Indeed, VSC-CDs exerted an alleviated effect on liver injury by chronic CCl_4_ injection.

The inflammatory response is involved in multicellular interaction and dynamically regulated by diverse factors covering the chronic irritants of viruses, drugs, and alcohol, which caused damage to hepatocytes. Hepatocyte injury inevitably contributed to the release of inflammatory cytokines from kupffer cells, thereafter activating HSCs. During an inflammatory response, various kinds of cytokines such as TNF-α, IL-1β, and IL-6 resulted in further damage to liver tissue (Li et al., 2015; Krenkel and Tacke, 2017). Serum TNF-α, IL-1β, and IL-6 concentrations in mice with CCl_4_-induced liver fibrosis were increased in our study, which coincided with the assay results of inflammatory factors in previous studies (Zhao et al., 2017; Abdelghffar et al., 2022). The role of VSC-CDs inhibited the expression of inflammatory mediators to regulate the inflammatory microenvironment within the liver and resist the activation of HSCs. Altogether, VSC-CDs exhibited anti-inflammation properties and alleviated some effects of chronic hepatic injury.

Oxidative stress is another indispensable factor related to liver fibrosis. In reference to previously discovered results (Brenner et al., 2013; Luedde et al., 2014), CCl_4_-induction gives rise to oxidative imbalance and oxidative stress damage. To our knowledge, the liver depends on defense mechanisms (antioxidant systems) to mitigate liver attack from reactive oxygen species (ROS); some antioxidants including SOD and GSH effectively scavenge free radicals (Han et al., 2017). Our results showed redox imbalance and decreased vitality of SOD and GSH occurred in CCl_4_-treated groups, which is consistent with similar liver fibrosis model (Zhang et al., 2018; Liu et al., 2021), while VSC-CDs could eliminate oxygen-free radicals to reduce oxidative stress damage. MDA, the product of liver oxidative mutilation and lipid peroxidation, was significantly raised in CCl_4_-induced groups, exerting severe damage to an antioxidant defense system. MDA content was greatly reduced after treatment with VSC-CDs. In conclusion, extenuating oxidative stress damage is a potential mechanism of anti-hepatic fibrosis in VSC-CDs treatment.

Exposure to CCl_4_ also provoked the activation of the TGF-β/Smad signaling pathway. The production of TGF-β_1_, as the main transforming growth factor isoform associated with liver fibrosis, facilitates transcription of Smad target genes (Paz and Shoenfeld, 2010). After the Smad signaling pathway was activated, phosphorylated Smad 2/3 complex binds with Smad_4_, which was translocated to the nucleus to regulate expression of target genes (Dooley et al., 2001). TGF-β_1_ and Smad3 levels were diminished via the modulation of VSC-CDs. Our study also validated VSC-CDs prevented the formation of ECM and aggravation of liver fibrosis. COI1 is one of the primary sources of excessive deposition of extracellular matrix materials, which forms fibrous scar tissues that replaces damaged normal tissue and ultimately compromises normal liver function (Li and Zhu, 2022). Notably, VSC-CDs reduced the production of COI1 in liver tissues. α-SMA, as a specific marker for HSCs activation, could be implicated in the TGF-β/Smad signaling pathway (Mao et al., 2015). α-SMA expressions was obviously upregulated under the stimulus of CCl_4_, indicating the activation and proliferation of HSCs, showing reduction after treatment of VSC-CDs. These results implied the inhibition effect of the TGF-β/Smad signaling may be a latent key mechanism in liver fibrosis.

In conclusion, we have developed an eco-friendly and safe carbon dot derived from traditional Chinese medicine that is characterized by an ultra-small particle size, distinctive fluorescence characteristics, and abundant functional groups on the surface. VSC-CDs have demonstrated the ability to partially reverse liver damage, inhibit inflammatory response and oxidative stress, and alleviate liver fibrosis. The potential mechanism involves the modulation of the TGF-β/Smad signaling pathway. These affirmative results suggest that VSC-CDs hold potential for clinical applications and exhibit promising prospects.

## Conclusion

In summary, novel fluorescent carbon dots were prepared with a green and eco-friendly method, and we conducted a comprehensive characterization of their morphological structure. They possessed desirable biocompatibility, low cytotoxicity, and high blood safety. Importantly, VSC-CDs demonstrated a mitigating effect on CCl_4_-induced liver fibrosis, as evidenced by the reduction in inflammatory cytokines (TNF-α, IL-6, IL-1β), elevation in antioxidant levels (SOD and GSH), and a decrease in lipid metabolites (MDA) to counteract oxidative stress damage. Furthermore, VSC-CDs demonstrated the ability to modulate the TGF-β/Smad signaling pathway and regulate the protein expressions of α-SMA and COI1, thereby inhibiting the progression of liver fibrosis. The above results highlight the underlying mechanism of action against liver fibrosis, laying a foundation for the development and application of carbon-based medicine. We propose that the safer and environmentally friendly VSC-CDs offer a promising avenue for the development of anti-liver fibrosis drugs.

## Data Availability

The original contributions presented in the study are included in the article/Supplementary material, further inquiries can be directed to the corresponding authors.
